# Supplementary material for: Antibody-drug conjugates with dual payloads for combating breast tumor heterogeneity and drug resistance
Source: Nat Commun. 2021 Jun 10;12:3528. doi: 10.1038/s41467-021-23793-7 (PMC8192907; doi:10.1038/s41467-021-23793-7)
Supplement: Supplementary file 3 — Reporting Summary [file 41467_2021_23793_MOESM3_ESM.pdf]

nature research

Corresponding author(s): Kyoji Tsuchikama

Last updated by author(s): May 7, 2021

Reporting Summary

Nature Research wishes to improve the reproducibility of the work that we publish. This form provides structure for consistency and transparency in reporting. For further information on Nature Research policies, see our Editorial Policies and the Editorial Policy Checklist.

Statistics

For all statistical analyses, confirm that the following items are present in the figure legend, table legend, main text, or Methods section.

n/a	Confirmed	
☐	☒	The exact sample size (*n*) for each experimental group/condition, given as a discrete number and unit of measurement
☐	☒	A statement on whether measurements were taken from distinct samples or whether the same sample was measured repeatedly
☐	☒	The statistical test(s) used AND whether they are one- or two-sided<br>*Only common tests should be described solely by name; describe more complex techniques in the Methods section.*
☒	☐	A description of all covariates tested
☐	☒	A description of any assumptions or corrections, such as tests of normality and adjustment for multiple comparisons
☐	☒	A full description of the statistical parameters including central tendency (e.g. means) or other basic estimates (e.g. regression coefficient) AND variation (e.g. standard deviation) or associated estimates of uncertainty (e.g. confidence intervals)
☐	☒	For null hypothesis testing, the test statistic (e.g. *F*, *t*, *r*) with confidence intervals, effect sizes, degrees of freedom and *P* value noted<br>*Give P values as exact values whenever suitable.*
☒	☐	For Bayesian analysis, information on the choice of priors and Markov chain Monte Carlo settings
☒	☐	For hierarchical and complex designs, identification of the appropriate level for tests and full reporting of outcomes
☒	☐	Estimates of effect sizes (e.g. Cohen's *d*, Pearson's *r*), indicating how they were calculated

Our web collection on statistics for biologists contains articles on many of the points above.

Software and code

Policy information about availability of computer code

Data collection	Kaluza acquisition software v2.1
Data analysis	Microsoft Excel 16, Prism 8, PKSolver version 2, MNova 10.02, FlowJo 10.6.1

For manuscripts utilizing custom algorithms or software that are central to the research but not yet described in published literature, software must be made available to editors and reviewers. We strongly encourage code deposition in a community repository (e.g. GitHub). See the Nature Research guidelines for submitting code & software for further information.

Data

Policy information about availability of data

All manuscripts must include a data availability statement. This statement should provide the following information, where applicable:
- Accession codes, unique identifiers, or web links for publicly available datasets
- A list of figures that have associated raw data
- A description of any restrictions on data availability

The authors declare that all data supporting the findings in this study are available within the paper, its supplementary information file, or from the corresponding author upon reasonable request.

Field-specific reporting

Life sciences study design

All studies must disclose on these points even when the disclosure is negative.

Sample size	Although no statistical analysis was performed prior to performing experiments, sample sizes were determined by following methods for similar experiments in the field reported previously by us and other groups (for example, Nat. Commun. 9, 2512 (2018), Mol. Cancer Ther. 17, 1494–1503 (2018), and Angew. Chem. Int. Ed. 56, 733–737 (2017)). These and other similar reports demonstrate the sample sizes are adequate to assure statistical power.
Data exclusions	One outlier from each of the following groups was excluded due to inconsistent readout:<br>Fig 3a: N297A trastuzumab, MMAF DAR 4 ADC, and MMAE DAR 6 ADC groups<br>Fig 3b: MMAE DAR 2 and MMAF DAR 2 ADC groups.<br>This was caused likely because these groups were placed on edge wells of 96-well plates.<br><br>No data or animals were excluded in in vivo studies.
Replication	All in vitro experiments were repeated as follows:<br>duplicate: cathepsin B cleavage<br>triplicate: cell-based ELISA, flow cytometry<br>quadruplicate: cell killing, clonogenicity, and inflammatory response<br>All attempts at replication were successful.<br><br>For in vivo studies, group assignments were as follows: n = 3 for MMAE DAR 4 ADC and n = 4 for other groups in PK study, n = 4 for tolerability study, n = 3 for blood chemistry analysis, n = 4-6 for JIMT-1/MDA-MB-231 admixed tumor model treatment, n = 11 or 12 for HCC1954-TDR tumor model treatment, and n = 3 for tumor targeting studies using dye conjugates. All animal studies were performed at once. We confirmed the validity and reproducibility of all studies except the blood chemistry analysis with statistical analysis.
Randomization	Samples/animals in all experiments were randomized to each group
Blinding	The investigators were not blinded to allocation during experiments

Reporting for specific materials, systems and methods

We require information from authors about some types of materials, experimental systems and methods used in many studies. Here, indicate whether each material, system or method listed is relevant to your study. If you are not sure if a list item applies to your research, read the appropriate section before selecting a response.

Materials & experimental systems

n/a	Involved in the study	
☐	☒	Antibodies
☐	☒	Eukaryotic cell lines
☒	☐	Palaeontology and archaeology
☐	☒	Animals and other organisms
☒	☐	Human research participants
☒	☐	Clinical data
☒	☐	Dual use research of concern

Methods

n/a	Involved in the study	
☒	☐	ChIP-seq
☐	☒	Flow cytometry
☒	☐	MRI-based neuroimaging

Antibodies

Antibodies used	Humanized mAbs (anti-HER2 and anti-TROP2 mAbs) were expressed in house (see Supplementary Notes). The other antibodies used in this study were purchased from commercial vendors as follows: Mouse anti-MMAE/F mAb (LEV-MAF3) from Levena Biopharma; goat anti-human IgG Fab–HRP conjugate (109-035-097), goat anti-human IgG Fc antibody (109-005-098), donkey anti-human IgG–HRP conjugate (709-035-149) and goat anti-mouse IgG-HRP conjugate (115-035-071) from Jackson ImmunoResearch; mouse anti-human ErbB-2 (CD340, HER2) Vio® Bright FITC (130-121-436) from Miltenyi Biotec; and rabbit anti-human HER2 mAb (2165S) from Cell Signaling. The dilutions used for each antibody are described in the Methods section of the manuscript.
Validation	Humanized anti-HER2 mAb (human IgG1 with murine sequences in the CDR regions): validated for purity and molecular weight by ESI-MS, HIC, and SEC analysis; validated in house for specificity and reproducibility by cell-based ELISA testing intact anti-HER2 mAb, anti-HER2 ADCs, and blank sample (no ADC control) in HER2-positive KPL-4 and HER2-negative MDA-MB-231 cells.<br>Humanized anti-TROP2 mAb (human IgG1 with murine sequences in the CDR regions): used only for drug conjugation, validated for purity and molecular weight by ESI-MS, HIC, and SEC analysis; not validated for antigen specificity.<br><br>Mouse anti-MMAE/F mAb (LEV-MAF3): used for ELISA as a detection antibody, validated in house for specificity, sensitivity, signal linearity, and reproducibility utilizing conjugates with or without MMAE or MMAF, blank sample (no ADC control), samples without a capture Ab, and serially diluted standards of each conjugate

goat anti-human IgG Fab–HRP conjugate (109-035-097): used for ELISA as a secondary Ab, validated in house for specificity, sensitivity, signal linearity, and reproducibility utilizing blank sample (no ADC control), samples without a capture Ab, and serially diluted standards of each conjugate
goat anti-human IgG Fc antibody (109-005-098): used for ELISA as a capture Ab, validated in house for specificity, sensitivity, signal linearity, and reproducibility utilizing blank sample (no ADC control), samples without a detection Ab, and serially diluted standards of each conjugate
donkey anti-human IgG–HRP conjugate (709-035-149): used for cell-based ELISA as a detection Ab, validated in house for specificity, sensitivity, signal linearity, and reproducibility utilizing blank sample (no ADC control) and samples without cells
goat anti-mouse IgG-HRP conjugate (115-035-071): used for ELISA as a tertiary antibody, validated in house for specificity, sensitivity, signal linearity, and reproducibility utilizing conjugates with or without MMAE or MMAF, blank sample (no ADC control), samples without a capture or secondary Ab, and serially diluted standards of each conjugate
mouse anti-human ErbB-2 (CD340, HER2) Vio® Bright FITC (130-121-436): used for flow cytometry, for QC and references see https://www.miltenyibiotec.com/US-en/products/erbb-2-cd340-antibody-anti-human-24d2.html#vio-bright-fitc:100-tests-in-200-ul
rabbit anti-human HER2 mAb (2165S): used for IHC, for QC and references see https://www.cellsignal.com/products/primary-antibodies/her2-erbb2-29d8-rabbit-mab/2165

Eukaryotic cell lines

Policy information about cell lines

Cell line source(s)

JIMT-1 (AddexBio), JIMT-1(MDR1+) (generated in house), HCC1954 (ATCC), HCC1954-TDR (generated in house), SKBR-3 (ATCC), THP-1 (ATCC), KPL-4 (provided by Dr. Junichi Kurebayashi at Kawasaki Medical School), MDA-MB-231 (ATCC), HepG2 (ATCC), and HEK293 (ATCC)

Authentication

ELISA for HER2 binding (KPL-4, MDA-MB-231), HER2-specific cell killing assay (JIMT-1, JIMT-1(MDR1+), HCC1954, HCC1954-TDR, SKBR-3, THP-1, KPL-4, MDA-MB-231, HepG2, and HEK293) and/or increased resistance to hydrophobic chemotherapeutic agents (JIMT-1, JIMT-1(MDR1+), HCC1954-TDR). THP-1 was purchased from ATCC and used without authentication

Mycoplasma contamination

All cell lines were periodically tested for mycoplasma contamination and tested negative.

Commonly misidentified lines
(See ICLAC register)

No such cell lines were used in this study

Animals and other organisms

Policy information about studies involving animals; ARRIVE guidelines recommended for reporting animal research

Laboratory animals

Female 6–8 weeks old CD-1® IGS mice (Charles River Laboratories, Strain Code: 022); Female BALB/cJ mice (5–6 weeks old, The Jackson Laboratory, Stock No: 000651); female NU/J mice (4–8 weeks old, The Jackson Laboratory, Stock No: 002019)

Wild animals

The study did not involve wild animals

Field-collected samples

The study did not involve samples collected from the field

Ethics oversight

All procedures were approved by either the Animal Welfare Committee of the University of Texas Health Science Center at Houston or the MD Anderson Cancer Center Institutional Care and Use Committee and performed in accordance with the institutional guidelines for animal care and use.

Note that full information on the approval of the study protocol must also be provided in the manuscript.

Flow Cytometry

Plots

Confirm that:

- [x] The axis labels state the marker and fluorochrome used (e.g. CD4-FITC).
- [x] The axis scales are clearly visible. Include numbers along axes only for bottom left plot of group (a 'group' is an analysis of identical markers).
- [x] All plots are contour plots with outliers or pseudocolor plots.
- [x] A numerical value for number of cells or percentage (with statistics) is provided.

Methodology

Sample preparation

HCC1954 and HCC1954-TDR cells were plated in a 6-well plate (3x10^5 cells/well) and incubated for 48 hours. Cells were harvested and resuspended in cold FACS buffer (PBS containing 1% FBS and 0.1% sodium azide) and then incubated with 2 µL of anti-ErbB-2 (CD340) Vio® Bright FITC (Cat. No.: 130-121-436, Miltenyl Biotec) for 20 min on ice. The cells were harvested, washed with cold FACS buffer three times and then analyzed using flow cytometry.

Instrument

Gallios Flow Cytometer (Beckman Coulter)

Software	Kaluza acquisition software v2.1 (Beckman Coulter) used for FACS data collection. FlowJo v10.6.1 (BD, USA) used for FACS data analysis.
Cell population abundance	No sorting was performed in this study
Gating strategy	DAPI was added into all samples for live cell selection. First, unstained HCC1954 cells were used to set up the scatter, single cell selection, live cell selections, and anti-HER2-FITC positive and negative areas. Next, IgG-FITC treated cells were used to confirm the specificity of anti-HER2-FITC. After confirming no non-specific binding of IgG-FITC, anti-HER2-FITC treated cells were applied to set up HER2-high, HER2-low, and HER2-negative areas. Total 10,000 cells were counted from each sample.

☒ Tick this box to confirm that a figure exemplifying the gating strategy is provided in the Supplementary Information.
